# Post-mortem diagnosis of COVID-19

**DOI:** 10.4102/ajlm.v10i1.1471

**Published:** 2021-03-24

**Authors:** Rujittika Mungmungpuntipantip, Viroj Wiwanitkit

**Affiliations:** 1 Private Practice, Bangkok, Thailand; 2 Department of Community Medicine, DY Patil University, Pune, India

## Abstract

We would like to share our impression on the report ‘Postmortem diagnosis of COVID-19: Antemortem challenges of three cases at the 37 Military Hospital, Accra, Ghana’.^1^ Attoh et al. concluded that ‘The outcome of COVID-19 testing is dependent on the sample type and accuracy of sampling amongst other factors’^1^ and suggested that ‘more autopsies are required to fully understand the pathogenesis of this disease in Ghanaians’.^1^ Indeed, post-mortem diagnosis of coronavirus disease 2019 (COVID-19) is possible and there are many reports of the existence of pathogenic viruses in autopsy specimens.^2,3^ Autopsy is also very useful for understanding the pathogenesis of this new disease. However, it must be performed with high caution. While there are no confirmed cases of the pathogen being spread from deceased patients, infection of forensic pathology workers has been reported.^4^ More autopsies might be recommended, but adequate biosafety and biosecurity, and other infection control precautions must be in place for these to occur.

## Response from Attoh et al.

In our article we did not go into details on the methodology. Although COVID-19 is a category 3 infectious agent, negative pressure systems are unavailable in our country. Therefore, a few modifications were made.

Post-mortems were performed using World Health Organization’s interim guidance for infection prevention and control for the safe management of a dead body in the context of COVID-19.^5^ The structural design of the autopsy suites used lacked negative pressure systems with filters. As a result, extractors were fitted to provide unidirectional airflow away from the anatomical pathology team into the atmosphere as an appropriate technology. Also, members of the anatomical pathology team were carefully selected to exclude those with chronic health conditions such as diabetes mellitus, and cardiovascular and respiratory diseases.

It is interesting to note that none of the forensic pathologists nor the anatomical pathology technicians involved in the study have shown COVID-19 symptoms up to today.
